# Development and internal validation of a spatiotemporal gait parameter-based diagnostic model for cerebral small vessel disease

**DOI:** 10.3389/fnagi.2026.1790471

**Published:** 2026-04-02

**Authors:** Yan-Yan Wang, He-Jao Mao, Ding-Ding Zhang, Zi-Ang Pan, Cheng-Qian Li, Zi-Yue Liu, Ming Yao, Jun Ni, Li-Xin Zhou, Bin Peng, Fei Han, Yi-Cheng Zhu

**Affiliations:** 1 Department of Neurology, Peking Union Medical College Hospital, Chinese Academy of Medical Sciences and Peking Union Medical College, Beijing, China; 2 Center for Prevention and Early Intervention, National Infrastructures for Translational Medicine, Institute of Clinical Medicine, Peking Union Medical College Hospital, Chinese Academy of Medical Science and Peking Union Medical College, Beijing, China

**Keywords:** cerebral small vessel disease, diagnostic model, gait disturbances, LASSO regression, nomogram

## Abstract

**Introduction:**

Cerebral small vessel disease (CSVD) is a common cause of functional decline in the elderly, yet its diagnosis often relies on neuroimaging, which may be inaccessible in routine practice. Given that gait impairment is a core feature of CSVD, we aimed to develop and validate a clinically applicable diagnostic model by integrating quantitative spatiotemporal gait parameters with conventional clinical features.

**Methods:**

This case–control study included 417 healthy controls from a community-based cohort and 117 hospital-based CSVD patients. Conventional clinical characteristics and quantitative spatiotemporal gait parameters were collected from all participants. A two-stage modeling approach was used, in which least absolute shrinkage and selection operator (LASSO) regression was first applied for predictor screening, followed by multivariable logistic regression for constructing the final diagnostic model. Model performance was assessed by discrimination (area under the curve [AUC]), calibration, and clinical utility (decision curve analysis [DCA]).

**Results:**

Six key variables were included in the final diagnostic model: sex, hypertension, body mass index (BMI), stride length, step frequency, and step width. The model exhibited excellent discrimination, achieving an AUC of 0.914 (95% CI: 0.886–0.943), along with strong calibration. DCA further confirmed its clinical utility, showing a greater net benefit across a wide range of threshold probabilities compared to default screening strategies.

**Conclusion:**

The diagnostic model developed in this study effectively identifies individuals at high risk of CSVD by leveraging quantitative spatiotemporal gait parameters alongside conventional clinical features.

## Introduction

Cerebral small vessel disease (CSVD) accounts for approximately 25% of ischemic strokes and is a major cause of cognitive decline, gait disturbance, and functional dependence in older adults, thereby imposing a significant burden on global healthcare systems (Han et al., 2021; Huang et al., 2025). Its structural markers on magnetic resonance imaging (MRI) include white matter hyperintensities (WMH), lacunes, and cerebral microbleeds (CMB) (Markus and de Leeuw, 2022). However, the insidious progression of CSVD often delays recognition until significant neurological deficits have developed (Hannawi et al., 2023).

Gait disturbance is a well-established clinical feature of CSVD, second only to cognitive impairment (Zhou et al., 2024). Therefore, gait analysis might serve as a tool to distinguish CSVD from normal aging. Nevertheless, in early-stage or subclinical CSVD, gait abnormalities are often subtle, phenotypically heterogeneous, and lack distinctive signs, making them challenging to identify through routine observation. In this context, quantitative gait parameters derived from multidimensional motor assessments have emerged as promising diagnostic biomarkers by objectively capturing specific CSVD-related alterations, such as shortened stride length and compensatory increases in cadence, which reflect underlying motor dysregulation (Mo et al., 2024).

Notwithstanding this potential, a significant translational gap persists. Current research remains predominantly focused on establishing correlations between gait metrics and neuroimaging findings, rather than advancing toward clinically applicable diagnostic models (Kim et al., 2016; van der Holst et al., 2018; Mao et al., 2023; Yang et al., 2025). Moreover, quantitative gait assessment has yet to be incorporated into standardized CSVD diagnostic protocols. Consequently, clinicians continue to rely on subjective visual inspection, which lacks the sensitivity required for detection of subclinical or early-stage disease.

To address these gaps, the present study aims to construct a diagnostic model based on quantitative spatiotemporal gait parameters and conventional clinical features, to enable early identification of individuals at high risk for CSVD. Those classified as high-risk by the model can be referred for further diagnostic confirmation via brain MRI, thereby optimizing the allocation of healthcare resources.

## Methods

### Study design and participants

This study employed a case–control design, with participants screened from two independent cohorts.

Patients with CSVD were recruited from the Neurology outpatient clinic of Peking Union Medical College Hospital (Mao et al., 2023). Specific inclusion criteria were as follows: (1) age > 40 years; (2) availability of complete cranial MRI data, with a total CSVD score ≥ 1 (Yang et al., 2025); (3) availability of complete quantitative gait parameters. Exclusion criteria included: (1) cerebral infarction due to large artery stenosis; (2) Parkinson’s disease, multiple sclerosis, intracranial tumors, or demyelinating diseases; (3) acute myocardial infarction or uncontrolled hypertension; (4) presence of aphasia, severe hearing or visual impairment; (5) severe cognitive decline or a diagnosis of dementia, which would prevent cooperation in the study. The study protocol was approved by the Medical Review Ethics Committee of PUMCH (reference number: JS-1280).

Healthy controls (HC) were drawn from the Shunyi Cohort, a prospective, community-based study investigating cardiovascular and age-related diseases (Cao et al., 2021; Han et al., 2021). Inclusion criteria included: (1) age ≥ 35 years; (2) availability of complete cranial MRI data, with a total CSVD score = 0; (3) availability of complete quantitative gait parameters. Exclusion criteria included: (1) having a history of stroke; (2) having a disease that severely affects gait, such as poliomyelitis, osteoarthritis, Parkinson’s syndrome; (3) having aphasia, severe hearing or vision impairment; (4) having a severely declined cognitive function or having been diagnosed with dementia and being unable to complete the study. The study was approved by the Ethical Committee of PUMCH (reference number: B-160).

According to the Declaration of Helsinki, all subjects signed an informed consent form. The participant selection process is illustrated in Supplementary Figure 1.

### Clinical assessment

Standardized questionnaires were used to collect demographic data and vascular risk factors (hypertension, diabetes, hyperlipidemia) for all participants. Body mass index (BMI) was calculated using weight and height as follows: BMI = weight (kg)/height^2^ (m^2^). Hypertension was defined as current use of antihypertensive medication, or a self-reported history of hypertension. Diabetes was defined as a self-reported history of diabetes or current use of oral hypoglycemic agents or insulin therapy. Hyperlipidemia was defined as self-reported history of hyperlipidemia or current use of lipid-lowering medication. Global cognitive function was assessed using the Mini Mental State Examination (MMSE) (Li et al., 2016).

### Gait measurement

Gait function was quantitatively assessed using a three-dimensional motion balance testing system (Ready Go, Beijing CAS-Ruiyi Information Technology Co., Ltd.) (Mao et al., 2023; Mo et al., 2024; Yang et al., 2025). This system employs cutting-edge AI technologies such as depth vision sensing, 3D human body reconstruction, and dynamic skeletal point tracking to perform high-precision motion capture in a compact and user-friendly device. While participants performed a three-meter walk test at their usual walking speed, the device recorded and digitally reconstructed their movements, enabling quantitative analysis of kinematic features and parameters through deep learning algorithms. Parameters included gait speed, stride length, step frequency, stride speed, swing speed, stance phase, swing phase, double-support phase, step height, step width, and turning time. Detailed definitions of the above-mentioned parameters are provided in Supplementary Table 1. Take the average of the left and right foot data for the same parameter as the final variable for analysis. To ensure measurement accuracy, automated annotations were manually verified and corrected when necessary.

### MRI acquisition and CSVD neuroimaging markers

Brain MRI was acquired on a 3-T Siemens Skyra scanner (Siemens, Germany). Four CSVD imaging markers—lacunes, CMB, WMH, and enlarged perivascular spaces (EPVS)—were assessed according to established STRIVE-2 criteria (Wardlaw et al., 2013), as detailed in our previous publications from the Shunyi cohort (Han et al., 2021; Wang et al., 2025) and CSVD cohort (Mao et al., 2023). Total CSVD score was quantified using an ordinal scale (range 0–4), with one point assigned for each of the following: presence of ≥1 lacune, presence of ≥1 CMB, moderate-to-severe basal ganglia EPVS (>10 spaces), severe periventricular hyperintensity (Fazekas grade 3) or confluent deep WMH (Fazekas grades 2–3) (Staals et al., 2014). The intra-rater agreement for both cohorts was assessed by trained physicians blinded to clinical data on random samples of 50 individuals with an interval of over 1 month between readings. The Shunyi cohort showed good intra-rater reliability as previously reported (Han et al., 2018; Zhai et al., 2018). High intra-rater consistency was also demonstrated in the CSVD cohort, with kappa coefficients of 0.82 for lacunes, 0.68 for white matter hyperintensities, and 1.00 for cerebral microbleeds.

### Sample size assessment

We applied the events-per-variable (EPV) rule to evaluate sample size adequacy for the prediction model, adhering to the empirically recommended minimum threshold of 10 EPV for Cox and logistic regression (Concato et al., 1995; Peduzzi et al., 1995, 1996).

### Statistical analysis

Missing values were imputed using the mean for continuous variables and the mode for categorical variables, respectively. Normality was assessed with histograms. All continuous variables were normally distributed, and are expressed as mean ± standard deviation (SD). Categorical variables are expressed as counts (percentages). Statistical comparisons between the CSVD group and healthy control group were performed using independent t-tests for continuous variables and chi-square tests for categorical variables. Differences were considered statistically significant at a bilateral *p-*value < 0.05.

A two-stage modeling approach was employed to construct the CSVD diagnostic model. First, the least absolute shrinkage and selection operator (LASSO) regression with 10-fold cross-validation was used for variable selection, applying the λ1se criterion. This approach effectively mitigated multicollinearity while preserving covariates with independent diagnostic value. The selected diagnostic variables identified by LASSO were subsequently incorporated into a multivariable binary logistic regression model. Backward stepwise selection was further employed, with only predictors with a *p*-value < 0.05 retained to generate the final diagnostic model.

Internal validation was conducted using bootstrap resampling with 1,000 iterations to estimate model optimism and evaluate stability of diagnostic factors. The validated core diagnostic factors were then visualized via a clinically applicable nomogram. Model performance was systematically evaluated in terms of discrimination (area under the receiver operating characteristic curve [AUC] with 95% confidence intervals [CIs]), calibration, and clinical utility (decision curve analysis [DCA]). All analyses were performed using the Statistical Program for Social Sciences (SPSS) statistical software (version 25; Chicago, IL, USA) and R Studio (v4.4.0).

## Results

### Participants characteristics

This study included 117 CSVD patients and 417 healthy controls. The CSVD group (mean age 64.37 ± 9.30 years; 65.0% male) exhibited significantly lower BMI and higher prevalence of hypertension, diabetes, and hyperlipidemia compared to controls (mean age 62.48 ± 7.46 years; 29.0% male). MMSE scores did not differ significantly between groups (CSVD: 26.23 ± 3.92 vs. healthy controls: 26.97 ± 3.68, *p* = 0.058).

**Figure 1:**
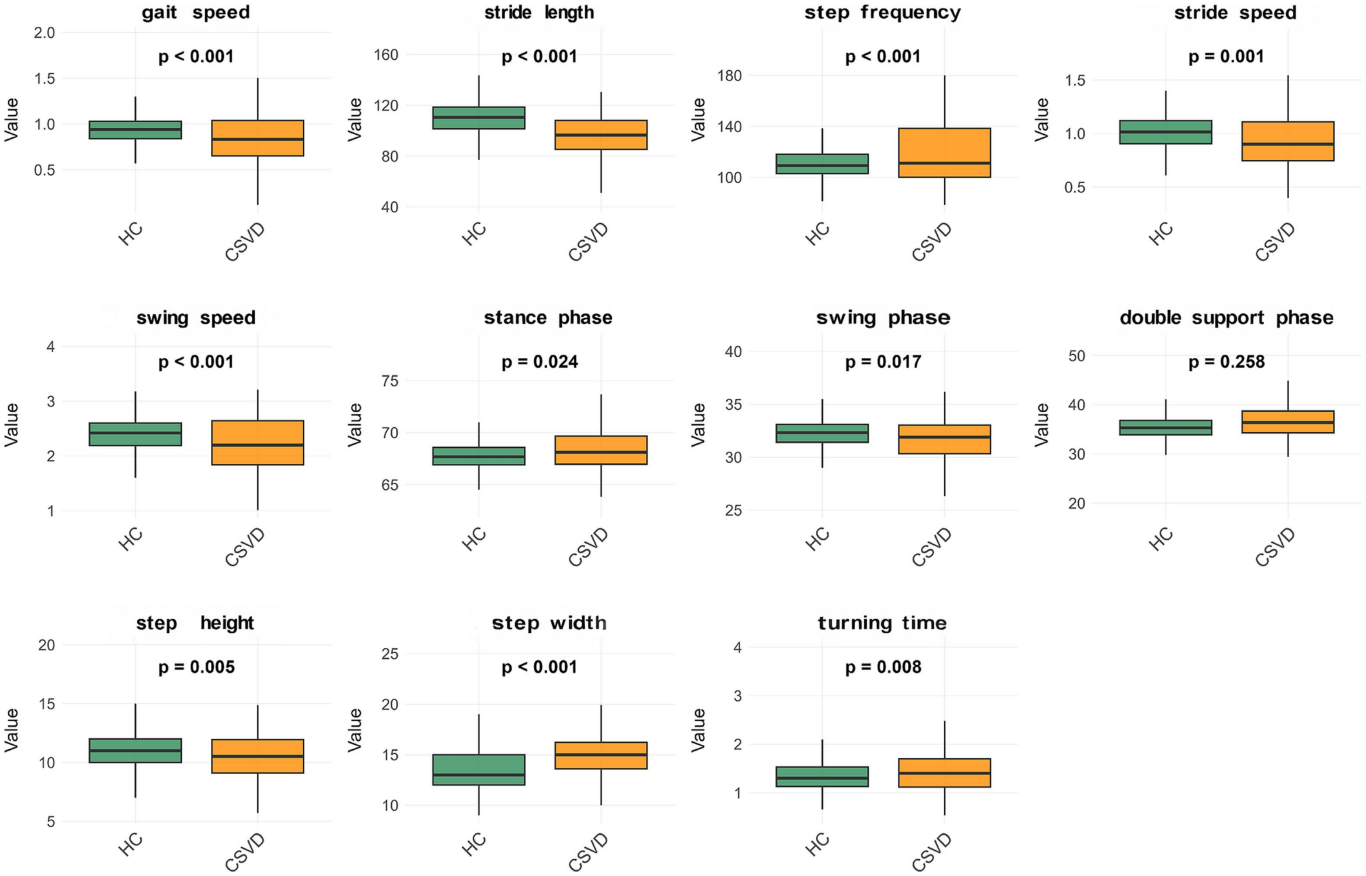
Difference of gait parameters in HC and CSVD groups. HC, Healthy control; CSVD, cerebral small vessel disease.

Compared with healthy controls, CSVD patients exhibited significant alterations across multiple gait domains. In terms of Speed-related parameters, patients demonstrated significantly slower performance, including reduced gait speed (0.84 ± 0.25 vs. 0.93 ± 0.15 m/s, *p* < 0.001), slower stride speed (0.93 ± 0.25 vs. 1.01 ± 0.16 m/s, *p* < 0.001), slower swing speed (2.22 ± 0.51 vs. 2.40 ± 0.31 m/s, *p* < 0.001), shorter stride length (93.15 ± 20.34 vs. 109.83 ± 13.48 cm, *p* < 0.001), and higher step frequency (121.13 ± 25.05 vs. 110.97 ± 11.48 steps/min, *p* < 0.001). Regarding Rhythm, the CSVD group showed a prolonged stance phase (68.20 ± 2.31 vs. 67.76 ± 1.43%, *p* = 0.049) and a reduced swing phase (31.76 ± 2.26 vs. 32.23 ± 1.43%, *p* = 0.035). However, no statistically significant difference was observed in the double-support phase (35.83 ± 4.85 vs. 35.34 ± 2.42%, *p* = 0.285). For Postural Control, patients displayed a wider step width (15.08 ± 2.40 vs. 13.51 ± 1.97 cm, *p* < 0.001) and a reduced step height (10.44 ± 2.01 vs. 11.00 ± 1.65 cm, *p* = 0.006). Finally, in the Turning domain, CSVD patients required significantly more time to complete turns (1.49 ± 0.53 vs. 1.34 ± 0.32 s, *p* = 0.003). This section only displayed the average gait parameters, calculated as the mean of bilateral measurements. To facilitate visual interpretation, a comparative analysis was provided in the form of box plots as shown in Figure 1, illustrating the differences between the CSVD and healthy control groups. Detailed unilateral data for the left or right feet were provided in Table 1.

**Table 1:** Characteristics of the study participants.

Variable	HC (*N* = 417)	CSVD (*N* = 117)	*p*
Clinical characteristics			
Age (year)	62.48 ± 7.46	64.37 ± 9.30	0.045*
Sex (male)	121 (29.0%)	76 (65.0%)	<0.001*
BMI (kg/m^2^)	26.68 ± 3.93	24.30 ± 3.38	<0.001*
Hypertension (yes)	113 (27.1%)	72 (61.5%)	<0.001*
Diabetes (yes)	27 (6.5%)	20 (17.1%)	<0.001*
Hyperlipidemia (yes)	62 (14.9%)	37 (31.6%)	<0.001*
MMSE (score)	26.97 ± 3.68	26.23 ± 3.92	0.058
Spatiotemporal gait parameter			
Gait speed (m/s)	0.93 ± 0.15	0.84 ± 0.25	<0.001*
Left stride length (cm)	110.01 ± 14.25	93.38 ± 20.73	<0.001*
Right stride length (cm)	109.66 ± 13.52	92.91 ± 20.35	<0.001*
Mean stride length (cm)	109.83 ± 13.48	93.15 ± 20.34	<0.001*
Left step frequency (steps/min)	111.73 ± 11.52	122.18 ± 25.50	<0.001*
Right step frequency (steps/min)	110.21 ± 12.27	120.09 ± 26.16	<0.001*
Mean step frequency (steps/min)	110.97 ± 11.48	121.13 ± 25.05	<0.001*
Left stride speed (m/s)	1.01 ± 0.16	0.92 ± 0.26	0.001*
Right stride speed (m/s)	1.01 ± 0.16	0.93 ± 0.26	0.001*
Mean stride speed (m/s)	1.01 ± 0.16	0.93 ± 0.25	<0.001*
Left swing speed (m/s)	2.40 ± 0.33	2.21 ± 0.52	<0.001*
Right swing speed (m/s)	2.41 ± 0.32	2.22 ± 0.53	<0.001*
Mean swing speed (m/s)	2.40 ± 0.31	2.22 ± 0.51	<0.001*
Left stance phase (%)	67.84 ± 1.82	68.41 ± 2.55	0.026*
Right stance phase (%)	67.68 ± 1.81	68.00 ± 2.71	0.226
Mean stance phase (%)	67.76 ± 1.43	68.20 ± 2.31	0.049*
Left swing phase (%)	32.15 ± 1.82	31.59 ± 2.55	0.027*
Right swing phase (%)	32.31 ± 1.81	31.94 ± 2.62	0.147
Mean swing phase (%)	32.23 ± 1.43	31.76 ± 2.26	0.035*
Double support phase (%)	35.34 ± 2.42	35.83 ± 4.85	0.285
Left step height (cm)	11.26 ± 1.98	10.64 ± 2.17	0.004*
Right step height (cm)	10.75 ± 1.97	10.24 ± 2.26	0.017*
Mean step height (cm)	11.00 ± 1.65	10.44 ± 2.01	0.006*
Step width (cm)	13.51 ± 1.97	15.08 ± 2.40	<0.001*
Turning time (s)	1.34 ± 0.32	1.49 ± 0.53	0.003*
Imaging characteristics			
Imaging markers of CSVD			
Lacune	0 (0.0%)	94 (80.3%)	<0.001*
CMB	0 (0.0%)	105 (89.7%)	<0.001*
PVH (Fazekas 3)	0 (0.0%)	85 (72.6%)	<0.001*
DWMH (Fazekas 2 and 3)	0 (0.0%)	104 (88.9%)	<0.001*
EPVS in basal ganglia (>10)	0 (0.0%)	74 (63.2%)	<0.001*
Total CSVD score			<0.001*
0	417 (100.00%)	0 (0.0%)	
1	0 (0.0%)	0 (0.0%)	
2	0 (0.0%)	23 (19.7%)	
3	0 (0.0%)	40 (34.2%)	
4	0 (0.0%)	54 (46.2%)	

HC, healthy control; CSVD, cerebral small-vessel disease; BMI, body mass index; MMSE, mini mental state examination. Mean = (Left +Right)/2; CMB, cerebral microbleed; PVH, periventricular hyperintensity; DWMH, deep white matter hyperintensity; EPVS, enlarged perivascular space.

*Statistically significant at *p* < 0.05 level, two-sided.

### Diagnostic model constructed based on LASSO-logistic regression

**Figure 2:**
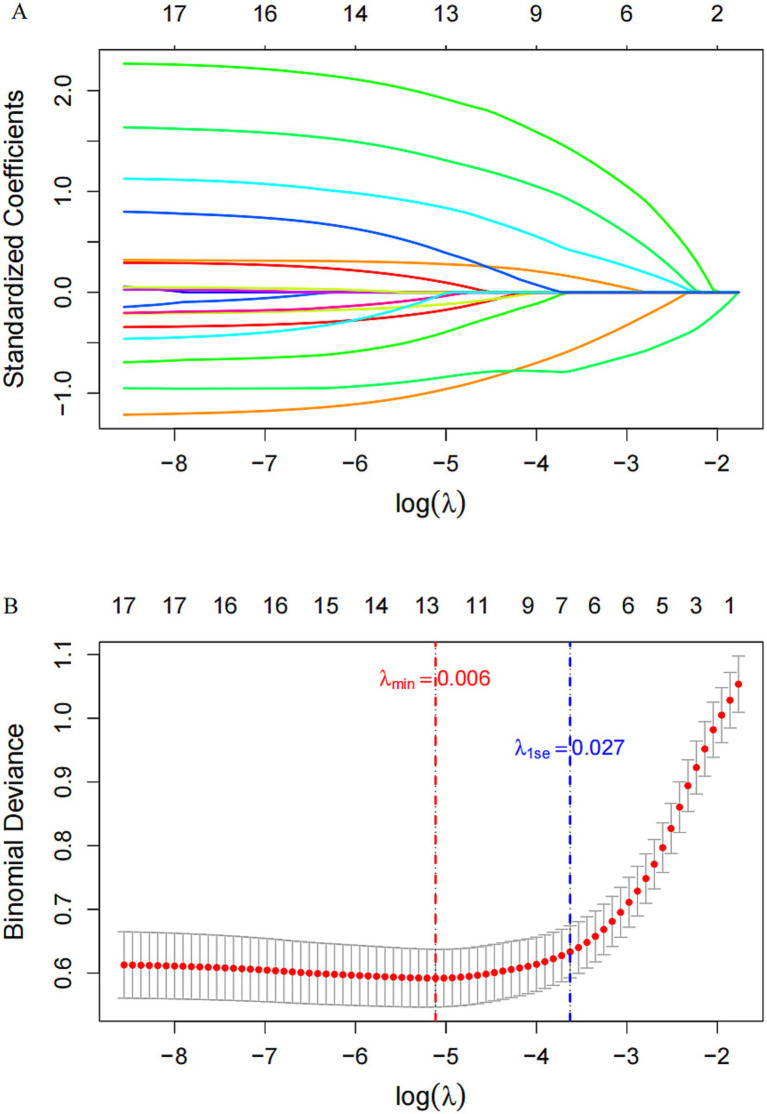
Screening of variables based on LASSO regression. **(A)** Coefficient variation profiles of candidate variables; **(B)** The process of optimal *λ* selection via cross-validation.

LASSO regression was used to screen potential diagnostic factors, including age, sex, BMI, MMSE, hypertension, diabetes, hyperlipidemia, and 11 gait parameters (gait speed, mean stride speed, mean swing speed, mean stride length, mean step frequency, mean step height, mean stance phase, mean swing phase, double-support phase, step width, and turning time). The coefficient profiles of these variables were shown in Figure 2A. The 10-fold cross-validation method was applied to the iterative analysis, and the optimal model with strong performance and the fewest variables was obtained at *λ* = 0.027 (Figure 2B). Based on the λ1se criterion, six variables including sex, BMI, hypertension, stride length, step frequency, and step width were selected to enter the next stage of modeling.

The six variables selected by the LASSO regression were incorporated into a multivariate binary logistic regression model, and the backward stepwise method was used for secondary variable screening. As presented in Table 2, six diagnostic factors independently related to CSVD were retained: male sex (Odds Ratio, OR = 9.16, 95% CI: 4.59–18.28, *p* < 0.001), BMI (OR = 0.75, 95% CI:0.68–0.82, *p* < 0.001), hypertension (OR = 4.99, 95% CI:2.66–9.36, *p* < 0.001), stride length (OR = 0.93, 95% CI:0.91–0.95, *p* < 0.001), step frequency (OR = 1.05, 95% CI:1.03–1.07, *p* < 0.001) and step width (OR = 1.20, 95% CI:1.02–1.41, *p* = 0.026). When these six variables were included together in the logistic regression model, all corresponding variance inflation factor (VIF) values were less than 2, well below the commonly used threshold of 5 or 10 for logistic regression, thus confirming the absence of significant multicollinearity. Based on these variables, the final diagnostic model for CSVD was constructed. To further visualize the relative impact of these six pivotal variables on CSVD diagnosis, we have included a corresponding forest plot as Supplementary Figure 2.

**Table 2:** Independent diagnostic factors for CSVD.

Variable	*β*	SE	*p* value	OR (95% CI)
Male sex	2.22	0.35	<0.001*	9.16 (4.59–18.28)
BMI	−0.29	0.05	<0.001*	0.75 (0.68–0.82)
Hypertension	1.61	0.32	<0.001*	4.99 (2.66–9.36)
Mean stride length	−0.07	0.01	<0.001*	0.93 (0.91–0.95)
Mean step frequency	0.05	0.01	<0.001*	1.05 (1.03–1.07)
Step width	0.18	0.08	0.026*	1.20 (1.02–1.41)

SE, standard error; OR, odds ratio; CI, confidence interval; BMI, body mass index.

*Statistically significant at *p* < 0.05 level, two-sided.

### Model performance evaluation and internal validation

**Figure 3:**
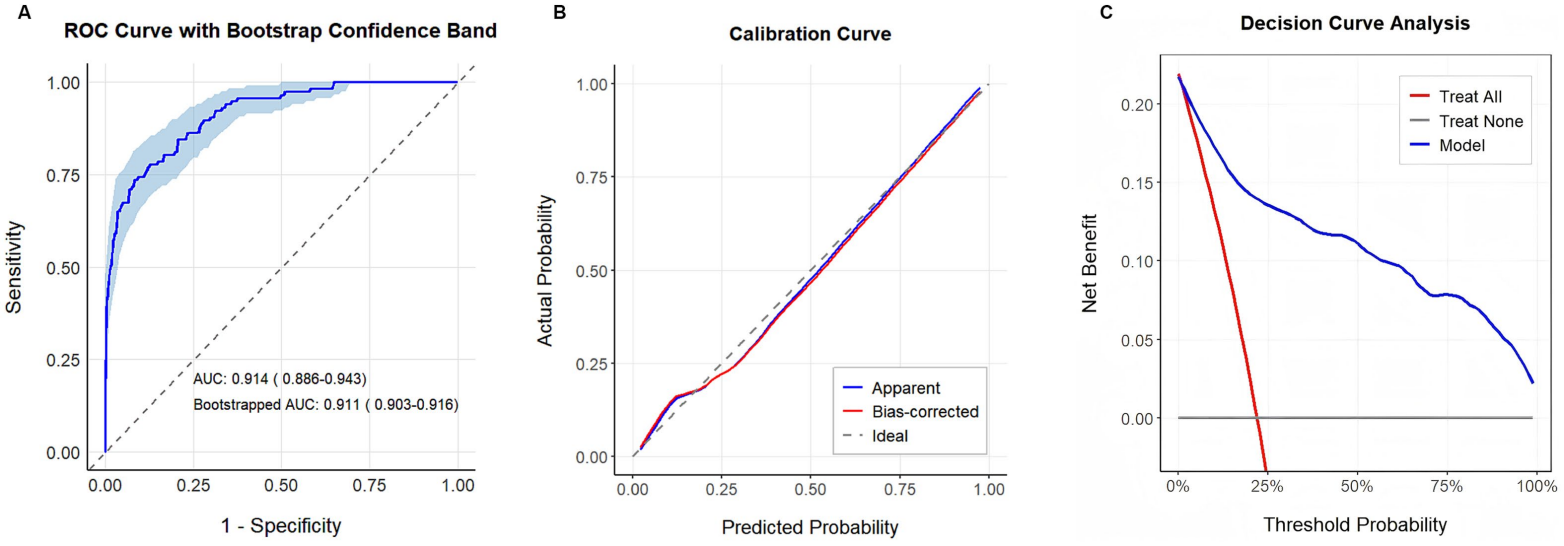
Performance evaluation of the diagnostic model for CSVD. **(A)** Bootstrap = 1,000, receiver operating characteristic curves of the model; **(B)** Bootstrap = 1,000, calibration curve of the model; **(C)** decision curve analysis of the model.

The model demonstrated excellent discrimination, with AUC of 0.914 (95% CI: 0.886–0.943). Internal validation via 1,000 bootstrap resamples maintained robust performance, yielding a corrected AUC of 0.911 (95% CI: 0.903–0.916) (Figure 3A).

Calibration analysis revealed strong agreement between predicted and observed CSVD risk. Bootstrap-corrected calibration curves closely aligned with the 45° reference line across the entire probability range (Figure 3B). Quantitative validation using the Brier score yielded a value of 0.085, indicating minimal deviation between predicted probabilities and actual outcomes.

The DCA (Figure 3C) further demonstrated the clinical utility of this model, indicating that across all the risk thresholds, it consistently provided greater net benefit than either “treat-all” or “treat-none” strategies. These results support the model’s potential for guiding individualized clinical decision-making in CSVD risk estimation.

### Model visualization

**Figure 4:**
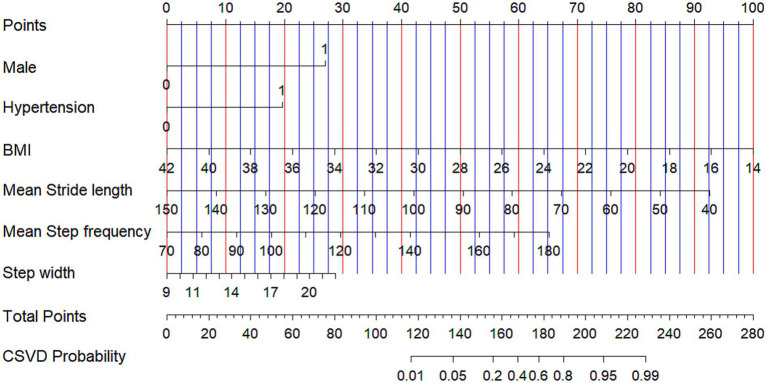
The nomogram used for diagnosing CSVD.

A nomogram was constructed to visualize the diagnostic model (Figure 4). Each diagnostic factor was assigned a “Points” value ranging from 0 to 100, displayed at the top of the nomogram. The sum of these points yielded a “Total Points” value, which corresponded directly to the predicted probability of CSVD. This tool allows individualized risk estimation in a user-friendly graphical format, supporting its potential clinical applicability.

## Discussion

This study developed a CSVD diagnostic model that combines conventional clinical features with quantitative spatiotemporal gait parameters. Six variables, including sex, history of hypertension, BMI, stride length, step frequency, and step width, were included in the final model. The model demonstrated excellent discrimination ability (AUC = 0.914) and calibration effect in differentiating CSVD patients from HC. It also showed consistent net benefits at different thresholds, highlighting its potential value in clinical applications. Through internal bootstrap validation, the model still exhibited good diagnostic efficacy (AUC = 0.911) and calibration performance. The diagnostic model was visualized using a nomogram, providing an objective auxiliary diagnostic tool for the early identification of CSVD.

Quantitative gait parameters demonstrated independent diagnostic value in this model, indicating that they can serve as a sensitive and effective objective indicator for identifying CSVD. Routine gait assessments, for example using three-dimensional motion analysis systems, could be implemented for individuals, particularly those with vascular risk factors. High-risk cases identified by the model could then by referred for further neurological evaluation and MRI confirmation, thereby facilitating earlier detection and intervention. However, the efficacy of this gait parameters depends on the introduction of quantitative motion function assessment tools. Traditional, semi-quantitative assessment methods based on experience may struggle to achieve the same diagnostic performance. As digital quantitative motion function assessment tools become increasingly prevalent in clinical practice and daily life, CSVD identification strategies based on quantitative motion parameters are expected to play an important role in primary health care systems.

CSVD primarily disrupts subcortical white matter and neural circuits critical for gait control, leading to a characteristic gait phenotype of reduced walking speed, shortened stride length, altered step frequency, and increased step width (de Laat et al., 2010a; Zhang et al., 2010; Smith et al., 2015; Su et al., 2022). For instance, a cross-sectional study provided evidence that microbleeds were independently associated with gait disturbances, particularly shorter stride length and impaired trunk balance, in elderly individuals with CSVD (de Laat et al., 2011). A study of 139 CSVD patients stratified by gait performance further confirmed that shortened stride length and widened base support reflect axonal demyelination and microstructural white matter disintegration (Zhao et al., 2022). Furthermore, studies have shown that lacunar infarcts involving the frontal lobe and thalamus were associated with lower gait speed, a relationship potentially mediated by the role of the frontal–basal ganglia–thalamic–cortical circuit (de Laat et al., 2010b). Our findings are consistent with these established characteristics, demonstrating shorter stride length, increased step frequency, and wider step width in CSVD patients compared with healthy controls. Unlike previous research that mainly reported correlations between gait abnormalities and CSVD neuroimaging markers (Su et al., 2017; Li et al., 2020; Yang et al., 2022), our study highlights the translational potential of incorporating quantitative gait analysis into routine diagnostic workflows, offering an objective and clinically practical tool for early CSVD detection and diagnosis.

Apart from gait parameters, some other findings also warrant further discussion. Old age is recognized as a risk factor for CSVD. The comparison between groups in this study also showed that the age of the CSVD group was significantly higher than that of the HC group, which is consistent with clinical experience. However, during the LASSO variable selection process, age was excluded due to collinearity with other variables such as hypertension and gait parameters. Therefore, it was not included in the final multivariate logistic regression model. The development of CSVD has been strongly linked to both the duration and cumulative exposure to hypertension, which represent critical modifiable risk factors, as established in a substantial body of studies (Blood Pressure Lowering Treatment Trialists’ Collaboration, 2014; Oparil et al., 2018). This pathogenic process is primarily driven by structural changes in the vascular wall, including thickening, luminal narrowing, and fibrinoid necrosis (Losso et al., 2024). Our findings further reinforce this robust association, aligning with existing evidence that underscores hypertension’s central role in CSVD pathogenesis. In addition, our findings indicated that male sex is associated with an increased susceptibility to CSVD, which aligns not only with the well-documented trend of higher risk of cardiovascular diseases in men (Connelly et al., 2021; Walli-Attaei et al., 2022; Swahn et al., 2024) but also with the male-predominant burden observed in certain CSVD markers (Yang et al., 2023). This observed disparity may be primarily attributable to estrogen’s vascular protection in women (Xue et al., 2013; Cao et al., 2025). Further mechanistic studies are needed to elucidate the specific pathophysiological pathways underlying these sex-based differences.

Surprisingly, our study found that healthy controls had a higher BMI than patients with CSVD. Two plausible explanations may account for this counterintuitive phenomenon. On one hand, control participants were recruited from rural communities in Beijing, where suboptimal dietary habits and lack of health awareness may contribute to a higher prevalence of obesity (NCD Risk Factor Collaboration, 2019; Wang et al., 2021). On the other hand, the relatively lower BMI of CSVD patients may be due to the reduction in salt and fat intake through a healthy diet after diagnosis. Notably, this inverse association between BMI and CSVD observed in our cohort is consistent with previous reports, such as a community-based study in southeastern China reporting that higher BMI was associated with a lower risk of CMB (OR: 0.95; 95% CI: 0.91–0.99) (Yang et al., 2023). Together, these observations suggest that BMI may serve as a proxy for underlying nutritional or metabolic determinants that influence CSVD risk in certain populations.

Several limitations should be acknowledged. Firstly, since our primary objective was to explore the diagnostic value of spatiotemporal gait parameters for CSVD, participants who did not complete gait assessments or brain MRI were excluded, which may introduce selection bias. Secondly, given that the diagnostic model was developed using a case–control design within a specific cohort having unique characteristics, its performance may decline when generalized to other settings with different populations or equipment, highlighting the need for external validation in future studies. Thirdly, it is important to note that gait abnormalities are not specific to CSVD and may also occur in other conditions such as orthopedic disorders and neurodegenerative diseases. Therefore, future multi-cohort studies involving diverse disease controls are warranted to enhance the model’s specificity and generalizability. Fourthly, this study only collected gait parameters under a single-task condition. In the future, it will be possible to conduct evaluations under a dual-task setting to better reflect the gait characteristics in real-world scenarios. Finally, although the present model performed well in identifying overall CSVD, it relies primarily on quantitative gait and basic clinical variables. Given the clinical heterogeneity of CSVD, most patients present with overlapping cognitive, motor, and emotional manifestations, whereas isolated phenotypes are relatively rare. Future models incorporating cognitive and emotional markers may further improve generalizability across the full phenotypic spectrum of CSVD.

## Conclusion

In conclusion, our study developed a high-performance diagnostic model for CSVD by integrating quantitative spatiotemporal gait parameters—specifically stride length, step frequency, and step width—with conventional clinical variables, highlighting the critical value of objective gait analysis in clinical assessment of CSVD. This model serves as a cost-effective, non-invasive tool to support an early screening strategy that directs expensive MRI confirmations to the highest-risk CSVD populations, thereby optimizing healthcare resource allocation. Further external validation through prospective, multicenter, large-scale studies will be essential to confirm its robustness and generalizability.

## Data Availability

The raw data supporting the conclusions of this article will be made available by the authors, without undue reservation.
