# Supplementary material for: Development and internal validation of a spatiotemporal gait parameter-based diagnostic model for cerebral small vessel disease
Source: Front Aging Neurosci. 2026 Apr 2;18:1790471. doi: 10.3389/fnagi.2026.1790471 (PMC13083152; doi:10.3389/fnagi.2026.1790471)
Supplement: Supplementary file 1 [file Data_Sheet_1.docx]

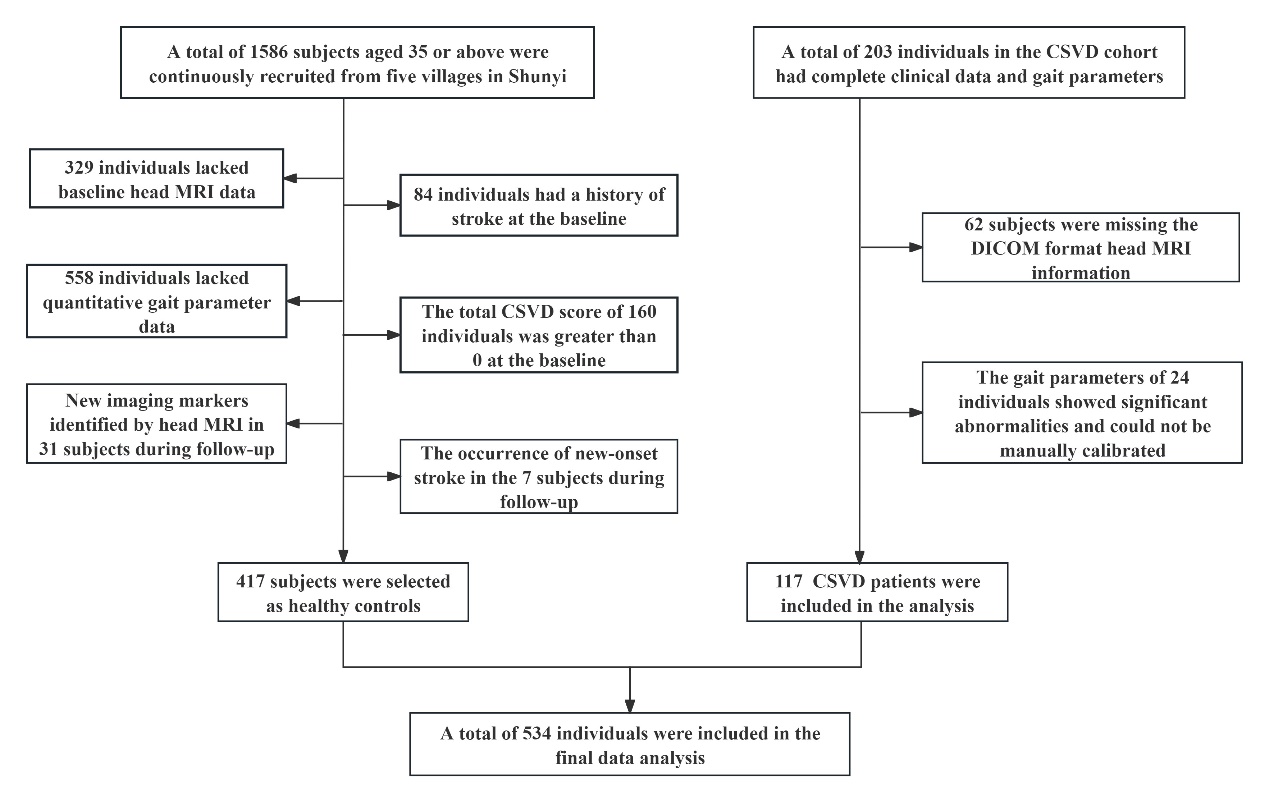


**Supplementary Figure 1**. The flowchart of the screening process for this study.


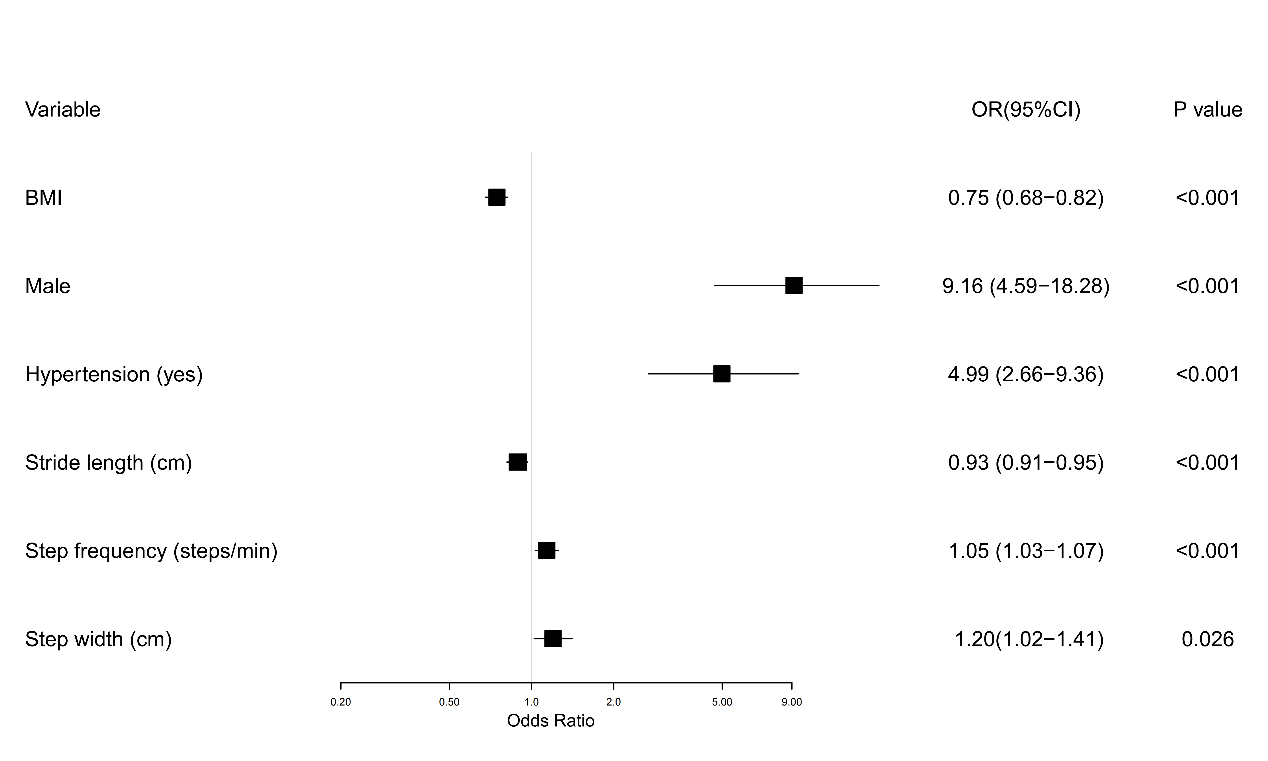


**Supplementary Figure 2**. Forest plot of six key CSVD diagnostic factors.

**Supplementary Table 1**. Definitions of gait parameters.

| **Parameter Category** | **Parameter Name** | **Unit** | **Definition** | **Differentiate Left/Right** |
| --- | --- | --- | --- | --- |
| **Speed** | Gait Speed | m/s | Average speed during the 3-meter walk test | No |
|  | Stride Length | cm | Distance between two consecutive footfalls of the same foot | Yes |
|  | Step Frequency | step/min | Number of steps taken per minute | Yes |
|  | Stride Speed | m/s | Stride length divided by stride time | Yes |
|  | Swing Speed | m/s | Swing distance divided by swing time | Yes |
| **Rhythm** | Stance Phase | % | Percentage of the gait cycle during which the foot is in contact with the ground | Yes |
|  | Swing Phase | % | Percentage of the gait cycle during which the foot is off the ground | Yes |
|  | Double Support Phase | % | Percentage of the gait cycle during which both feet are in contact with the ground | Yes |
| **Postural Control** | Step Width | cm | Lateral distance between the two feet during walking | No |
|  | Step Height | cm | Maximum vertical distance of the foot during the swing phase | Yes |
| **Turning** | Turning Time | s | Time taken to complete a turn | No |

**Supplementary Table 2. Association of Gait Parameters with CSVD in Different Adjusted Models**

|  | **Model1** |  | **Model2** |  | **Model3** |  |
| --- | --- | --- | --- | --- | --- | --- |
| **Variable** | **OR (95%CI)** | ***P* value** | **OR (95%CI)** | ***P* value** | **OR (95%CI)** | ***P* value** |
| Mean stride length | 0.95 (0.94–0.97) | <0.001* | 0.92 (0.90–0.94) | <0.001* | 0.93 (0.91–0.95) | <0.001* |
| Mean step frequency | 1.04 (1.03–1.06) | <0.001* | 1.05 (1.03–1.07) | <0.001* | 1.05 (1.03–1.07) | <0.001* |
| Step width | 1.29 (1.15–1.46) | <0.001* | 1.19 (1.01–1.39) | 0.03* | 1.19 (1.01–1.40) | 0.03* |

Note:

Model 1: Unadjusted model.

Model 2: Adjusted for age, sex, and BMI.

Model 3: Further adjusted for hypertension, diabetes mellitus, hyperlipidemia, and MMSE based on Model 2.

Model 1 (Crude Model): Only three core gait metrics (mean stride length, mean step frequency, step width) were included without adjusting for any confounders. All gait parameters showed significant associations with CSVD (all *P* < 0.001), preliminarily indicating the predictive potential of gait characteristics.

Model 2 (Demographic Confounder Adjusted Model): On the basis of Model 1, we further adjusted for age, sex, and BMI—key demographic factors highlighted by the reviewer. After controlling for these variables, all gait metrics remained statistically significant (mean stride length: OR=0.92, 95%CI: 0.90–0.94, *P*<0.001; mean step frequency: OR=1.05, 95%CI: 1.03–1.07, *P*<0.001; step width: OR=1.19, 95%CI: 1.01–1.39, *P*=0.03), with no substantial attenuation in effect sizes compared with Model 1.

Model 3 (Full Clinical Confounder Adjusted Model): We additionally adjusted for hypertension, diabetes, hyperlipidemia, and MMSE score on the basis of Model 2, to account for potential interference from comorbidities and cognitive function. Notably, the significant associations of all gait parameters with CSVD were still preserved (mean stride length: OR=0.93, 95%CI: 0.91–0.95, *P*<0.001; mean step frequency: OR=1.05, 95%CI: 1.03–1.07, *P*<0.001; step width: OR=1.19, 95%CI: 1.01–1.40, *P*=0.03), and the OR values and 95% confidence intervals remained highly consistent across the three models.

The stability of the effect size and statistical significance of gait parameters in all hierarchical models clearly indicates that stride length, step frequency, and step width have independent diagnostic value for the identification of CSVD.
